# Distinguishing Tachycardia Mediated From Structural Cardiomyopathy: Association of Late Gadolinium Enhancement With Recovery of Ventricular Systolic Function Following Atrial Fibrillation Ablation

**DOI:** 10.1161/JAHA.116.003292

**Published:** 2016-09-26

**Authors:** Saman Nazarian

**Affiliations:** ^1^ Section for Cardiac ElectrophysiologyHospital of the University of PennsylvaniaUniversity of Pennsylvania Perelman School of MedicinePhiladelphiaPA

**Keywords:** Editorials, arrhythmia, atrial fibrillation, cardiac magnetic resonance, catheter ablation, scar, Arrhythmias

## Introduction

A common clinical scenario in electrophysiology practice involves patients with arrhythmia and left ventricular (LV) dysfunction. The concept that atrial fibrillation (AF) or other arrhythmias can induce cardiomyopathy in the absence of organic heart disease has been known since the 1950s.^1^, ^2^, ^3^, ^4^ In the case of premature ventricular contractions, there is now substantial evidence to support a role for arrhythmia suppression by ablation for subsequent recovery of LV function.^5^, ^6^, ^7^, ^8^, ^9^ Similarly, it is recognized that, at least in subsets of patients with systolic congestive heart failure (CHF) and AF, LV function can improve by rate^10^ or rhythm control.^11^, ^12^, ^13^ In the overall population of patients with AF and CHF, however, rhythm control with medications and serial cardioversions does not appear to reduce the rate of death from cardiovascular causes, and increases hospitalizations when compared with a rate‐control strategy.^14^ Yet, in a small study comparing AF ablation versus atrioventricular‐node ablation with biventricular pacing in patients with CHF and drug‐refractory AF, those randomized to ablation had improved quality of life, longer 6‐minute walks, and improved LV function at 6 months.^15^ These seemingly incongruent results are not surprising. The association of AF with CHF may follow any of the following scenarios depending on the individual patient: (1) AF in isolation is causal for CHF; (2) AF is on the causal pathway for CHF as a mediator; (3) AF is 1 of 2 or more causal agents for CHF; (4) both AF and CHF are caused by a third factor that affects the underlying atrial and ventricular myocardium; and (5) AF is a result of the suboptimal atrial hemodynamics caused by LV dysfunction and CHF. It is easy to envision that the greatest benefit for AF suppression in the setting of CHF would be observed in the first and second scenarios. Therefore, given the attendant potential complications of AF ablation,^16^, ^17^ proper patient selection is of utmost importance. Appropriate patient selection is particularly important in patients with persistent or long‐standing persistent AF and LV dysfunction, where the risk/benefit ratio must be carefully scrutinized.

In this issue of the *Journal of the American Heart Association*, Addison et al present valuable results to aid patient selection for AF ablation in the setting of LV dysfunction.^18^ The researchers retrospectively identified 172 patients with LV dysfunction on cardiac magnetic resonance (CMR) performed before AF ablation. Among all patients, the median time between first symptomatic AF diagnosis and ablation was 30 months (range, 0.8–7.0 years), and 30% presented with paroxysmal and 70% with persistent AF. Of 172 patients, 25% had LV late gadolinium enhancement (LGE) on CMR. Of the patients with LV LGE, 23% had ischemic transmural infarct, 35% ischemic subendocardial infarct, 35% mid‐myocardial nonischemic, and 7% right ventricular insertion‐site LGE patterns. The median LV ejection fraction was 43%, with a range of 20% to 49%, and the majority of patients were on optimal medical therapy for LV dysfunction. During follow‐up after ablation, 40% and 38% presented with early (median, 1.5 months) and late (median, 9 months) AF recurrence, respectively. Admissions for heart failure were documented in 5%, and 13% died. The median change in LV ejection fraction among all patients was +7% (25th–75th percentiles of change: −1% to +14%). Of all patients, 53% had recovery of LV ejection fraction to >50% by 42 months following ablation. Upon multivariable analysis, the presence of LV LGE at baseline (odds ratio, 0.01) was inversely associated with recovery of LV dysfunction. Notably, the presence of LV LGE (hazard ratio, 3.3) was independently associated with mortality in a multivariable model including recovery of LV function.

The researchers have discussed limitations, including possible underestimation of AF recurrence attributed to the retrospective design as well as the lack of comparison to randomized controls treated with medical therapy. Baseline measurements were performed by CMR; however, LV function following ablation was measured by echocardiography in 77% of patients. This is mitigated by the fact that in the 20% of patients with both echocardiography and CMR follow‐up, the measurement bias was minimal (mean, 2% lower by echocardiography), with an excellent overall correlation (*r*=0.95; *P*<0.001). A small possibility for differential bias has not been excluded. Echocardiography or CMR measurements may be biased upward or downward depending upon the rhythm and/or the presence of LV dysfunction at the time of measurement. Such a differential bias would be missed in an analysis that compared the 2 modalities in a subcohort with persistent LV dysfunction and recurrent AF. Optimal medical therapy was used in the majority of patients, but the duration of optimal CHF therapy and rate control before ablation is unknown. Therefore, part of the LV function recovery may be attributable to rate and CHF control therapy before ablation. Also, the direction of association between ablation and recovery of LV function in the absence of LGE is unknown. Improved hemodynamics and LVEF with medical therapy in the absence of LGE may have increased the likelihood of rhythm control following AF ablation. On the other hand, the absence of LGE may have led to improved rhythm control attributed to AF ablation and led to subsequent improvement in hemodynamics and LVEF.

Nevertheless, the article provides important data for the care of a substantial portion of patients with LV dysfunction. Addison et al's data suggest that an aggressive rhythm control strategy utilizing AF ablation appears to associate with relatively favorable odds for recovery of LV function, reduced heart failure hospitalizations, and reduced mortality in patients without baseline LV LGE. Therefore, the study tips the risk/benefit balance of AF ablation in patients with AF and LV dysfunction without LV LGE toward benefit. Future prospective, randomized studies of rate versus rhythm control in AF patients with and without LGE are warranted to further refine these important results.

## Disclosures

Dr Nazarian is a scientific advisor to CardioSolv, St Jude medical, and Biosense Webster as well as a principal investigator for research funding from Biosense Webster, Inc. Additionally, Dr Nazarian receives funding from the National Institutes of Health (R01HL116280). The views expressed in this document reflect the opinions of the author and do not necessarily represent the official views of the National Institutes of Health or the National Heart, Lung and Blood Institute.
